# Association of the *NCAN-TM6SF2-CILP2-PBX4-SUGP1-MAU2* SNPs and gene-gene and gene-environment interactions with serum lipid levels

**DOI:** 10.18632/aging.103361

**Published:** 2020-06-22

**Authors:** Guo-Xiong Deng, Rui-Xing Yin, Yao-Zong Guan, Chun-Xiao Liu, Peng-Fei Zheng, Bi-Liu Wei, Jin-Zhen Wu, Liu Miao

**Affiliations:** 1 Department of Cardiology, Institute of Cardiovascular Diseases, The First Affiliated Hospital, Guangxi Medical University, Nanning 530021, Guangxi, People’s Republic of China; 2 Guangxi Key Laboratory Base of Precision Medicine in Cardio-cerebrovascular Disease Control and Prevention, Nanning 530021, Guangxi, People’s Republic of China; 3 Guangxi Clinical Research Center for Cardio-cerebrovascular Diseases, Nanning 530021, Guangxi, People’s Republic of China; 4 Department of Cardiology, Liuzhou People’s Hospital, Liuzhou 545006, Guangxi, People’s Republic of China

**Keywords:** single nucleotide polymorphism, gene-gene interactions, gene-environment interactions, generalized multifactor dimensionality reduction, lipid level

## Abstract

This study investigated the association of the *NCAN-TM6SF2-CILP2-PBX4-SUGP1-MAU2* SNPs and gene-gene and gene-environment interactions with serum lipid levels in the population of Southwest China. Genotyping of 12 SNPs (i.e., rs2238675, rs2228603, rs58542926, rs735273, rs16996148, rs968525, rs17216525, rs12610185, rs10401969, rs8102280, rs73001065 and rs150268548) was performed in 1248 hyperlipidemia patients and 1248 normal subjects. The allelic and genotypic frequencies of the detected SNPs differed substantially between the normal and hyperlipidemia groups (*P* < 0.05-0.001), and the association of the 12 SNPs and hyperlipidemia was also observed (*P* < 0.004-0.0001). Four haplotypes (i.e., *NCAN* C-C, *CILP2* G-T, *PBX4-SUGP1* G-C, and *MAU2* C-A-G-T) and 5 gene-gene interaction haplotypes (i.e., rs2238675C-rs2228603C, rs16996148G-rs17216525T, rs12610185G-rs10401969C, rs73001065G-rs8102280A-rs150268548G-rs968525C and rs73001065C-rs8102280A-rs150268548G-rs96852）showed a protective effect, whereas four other haplotypes (i.e., *TM6SF2* T-A, *TM6SF2* C-A, *MAU2* G-G-G-C and *MAU2* C-G-A-T), as well as 4 gene-gene interaction haplotypes (i.e., rs58542926C-rs735273A, rs58542926T-rs735273A, rs73001065G-rs8102280G-rs150268548G-rs968525C, and rs73001065C-rs8102280G-rs150268548A-rs968525T), exhibited an inverse effect on hyperlipidemia (*P* < 0.05-0.0001). There were notable three-locus models comprising SNP-SNP, SNP-environment, and haplotype-haplotype interactions (*P* < 0.05-0.0001). The individuals with some genotypes and haplotypes reduced the prevalence of hyperlipidemia, whereas the individuals with some other genotypes and haplotypes augmented the prevalence of hyperlipidemia. The *NCAN-TM6SF2-CILP2-PBX4-SUGP1-MAU2* SNPs and gene-gene and gene-environment interactions on hyperlipidemia were observed in the population of Southwest China.

## INTRODUCTION

Cardiovascular diseases (CVDs) have been a notable cause of disability and death worldwide. Numerous epidemiological, clinical and experimental studies have demonstrated that hyperlipidemia is involved in the progression of atherosclerosis which, in turn, causes CVD. Hyperlipidemia has a vital function in the onset of atherosclerosis; it can lead to oxidative stress and chronic inflammation and induce damage to macromolecules, endothelial cell apoptosis, proliferation and migration of vascular smooth muscle cells, all of which involve the formation of atheroma, leading to the development of atherosclerosis [1]. Although people strive to change their lifestyle and take such medications as statins and other lipid-lowering drugs, the incidence of CVD is still increasing [2]. It is difficult for many individuals to reach standard serum lipid levels even after taking medications, or some of them may suffer from certain side effects [3]. Thus, it is essential to discover variants for new markers that regulate serum lipid profiles, which may facilitate efforts to further improve hyperlipidemia and thus may reduce the probability of CVD.

Recently, genome-wide association studies (GWAS) have identified numerous new loci at chromosome 19p13 that can modify lipid metabolism, such as the neurocan gene (*NCAN*, Gene ID:1463, OMIM: 600826), transmembrane 6 superfamily member 2 gene (*TM6SF2*, Gene ID: 53345, OMIM: 606563), cartilage intermediate layer protein 2 gene (*CILP2*, Gene ID:148113, OMIM: 612419), PBX homeobox 4 gene (*PBX4*, Gene ID:80714, OMIM: 608127), SURP and G-patch domain containing 1 gene (*SUGP1*. Gene ID:57794, OMIM: 607992, formerly known as F23858, RBP, SF4), and MAU2 sister chromatid cohesion factor gene (*MAU2*, Gene ID:23383, OMIM: 614560) [4, 5]. These loci are located in regions associated with morbidity due to coronary artery diseases (CAD) [6–8]. Kozlitina et al. [9] demonstrated that hepatic triglyceride content (HTGC) was associated with *TM6SF2* (rs58542926 c.449 C > T). Several *in vitro* experiments have found that knockdown of *TM6SF2* can cause decreased synthesis of apolipoprotein (Apo) B and triglyceride (TG)-rich lipoproteins [9, 10]. Moreover, *TM6SF2* knockdown also causes accumulation of cellular TG, which has been reported as a significant increase in the number and size of lipid droplets at the subcellular level [11]. In contrast, overexpression of *TM6SF2* can result in a reduction in the number and size of lipid droplets [11]. Some previous studies have established that the 19p13.11 locus linked together the adjoining genes *NCAN* and *PBX4* with dyslipidemia, and these relationships may now be attributed to *TM6SF2* [12–14]. Rašlová et al. [15] established that there was an association between *CILP* polymorphism and esterification rate of cholesterol in plasma high-density lipoprotein and affect lipid metabolism. Zhou et al. [8] identified that the rs16996148 SNP in *NCAN-CILP* was significantly associated with reduced CAD risk in the Chinese populations. Luptakova et al. [16] established that the minor T allele of *CILP2* can fight against the elevation of lipid and lipoprotein in serum. Some reports have also documented that after transfecting the HepG2 and Huh7 cell lines with siRNAs for *SUGP1*, the transcript concentrations and protein levels of *SUGP1* were reduced by 45–70% and 72–91%, respectively [17]. Moreover, overexpression of *SUGP1* was correlated with greater elevation in total cholesterol (TC) and TG, both *in vivo* and *in vitro* [17]. In addition, overexpression of *SUGP1* led to greater activity of hepatic 3-hydroxy-3-methylglutaryl coenzyme A (HMG CoA) reductase (HMGCR) enzyme, but there was no change in the transcript level of hepatic HMGCR [17]. *MAU2*, which is located close to *NCAN* on chromosome 19, has been identified to have an association with TC, low-density lipoprotein cholesterol (LDL-C) and TG in serum [18, 19].

The causes of these variations have not been fully elucidated, but hyperlipidemia is considered to be a complex disease characterized by subtle interpatient variability, comprising host genetic factors and environmental interactions that generate disease phenotypes and establish disease advancement. Although a series of studies have revealed that environmental factors have determined the presence of dyslipidemia [20–22], it is also known that genetic factors have a vital role and can establish how an individual responds to challenges [6, 12]. Our previous study established that the *BCL3-PVRL2-TOMM40* SNPs were located on chromosome 19 p11, the prevailing model of rs157580 and rs8100239 SNPs, and some haplotypes and gene-gene interaction haplotypes were involved in protection, although other haplotypes and gene-gene interaction haplotypes, including the prevailing model of rs6859, rs3810143, rs519113 and rs10402271 SNPs, indicated an augmented morbidity function [23]. Even though we have conducted substantial research and made extensive progress in identifying genetic modifiers, the relationship between hyperlipidemia and other gene polymorphisms has not been fully elucidated. In this study, we focus on the association of the *NCAN*, *TM6SF2*, *CILP2*, *PBX4*, *SUGP1* and *MAU2* single nucleotide variants, gene-environment interactions and gene-gene interactions with serum lipid levels. Configurations of the relationships among SNPs throughout the genome might be categorized with regard to linkage disequilibrium (LD) and haplotype [24].

## RESULTS

### Demographic and biochemical characteristics

Table 1 describes the typical characteristics of 2,496 participants from both groups. Systolic blood pressure, diastolic blood pressure, pulse pressure, TC, TG, high-density lipoprotein cholesterol (HDL-C) and LDL-C levels were substantially higher in hyperlipidemia than in normal groups (*P* < 0.05-*P* < 0.001 for all), whereas body weight, waist circumference, and blood glucose levels were significantly lower in hyperlipidemia than in normal groups (*P* < 0.001 for all). However, there was no substantial difference in age, sex ratio, height, body mass index (BMI), smoking status, alcohol consumption, ApoA1, ApoB levels, or the ApoA1/ApoB ratio between the two groups (*P* > 0.05 for all).

**Table 1:** Comparison of demographic, lifestyle characteristics and serum lipid levels between the normal and hyperlipidemia groups.

**Parameter**	**Normal**	**Hyperlipidemia**	***t* (*x*^2^)**	***P***
Number	1248	1248		
Male/female	478/770	487/761	0.137	0.742
Age (years)^1^	55.98±12.78	56.87±12.12	1.672	0.205
Height (cm)	154.02±7.74	153.53±8.07	2.495	0.114
Weight (kg)	53.01±8.92	52.95±10.60	23.359	2E-006
Body mass index (kg/m^2^)	22.31±3.22	22.36±3.70	3.630	0.057
Waist circumference	77.13±7.81	76.34±9.21	24.311	2E-007
Smoking status [*n* (%)]				
Non-smoker	936(75.00)	984(78.84)		
≤ 20 cigarettes/day	276(22.11)	233(18.66)		
> 20 cigarettes/day	36(2.89)	30(2.40)	5.378	0.068
Alcohol consumption [*n* (%)]				
Non-drinker	1007(80.69)	994(79.65)		
≤ 25 g/day	121(9.66)	136(10.90)		
> 25 g/day	120 (9.65)	118(9.45)	0.997	0.614
Systolic blood pressure (mmHg)	129.26±19.28	135.89±24.76	69.976	2E-016
Diastolic blood pressure (mmHg)	81.55±11.46	83.47±12.55	12.250	E-005
Pulse pressure (mmHg)	47.71±15.29	52.42±18.56	50.587	4E-015
Glucose (mmol/L)	6.18±1.91	6.15±1.43	21.278	E-006
Total cholesterol (mmol/L)	4.97±1.05	5.21±1.09	6.203	0.012
Triglyceride (mmol/L)^2^	1.49(0.68)	1.63(0.71)	7.036	0.005
HDL-C (mmol/L)	1.75±0.50	1.81±0.60	12.497	2E-005
LDL-C (mmol/L)	2.88±0.85	2.99±0.79	6.198	0.017
ApoA1 (g/L)	1.35±0.26	1.39±0.32	0.361	0.548
ApoB (g/L)	0.84±0.19	0.88±0.20	1.484	0.223
ApoA1/ApoB	1.67±0.50	1.66±0.57	0.095	0.758

HDL-C: high-density lipoprotein cholesterol. LDL-C: low-density lipoprotein cholesterol. Apo: Apolipoprotein. ^1^Mean ± SD determined by *t*-test. ^2^Because the data were not normally distributed, the value of triglyceride was presented as median (interquartile range), and the difference between the two groups was determined by the Wilcoxon-Mann-Whitney test.

### Genotypic and allelic frequencies in both groups

**Figure 1:**
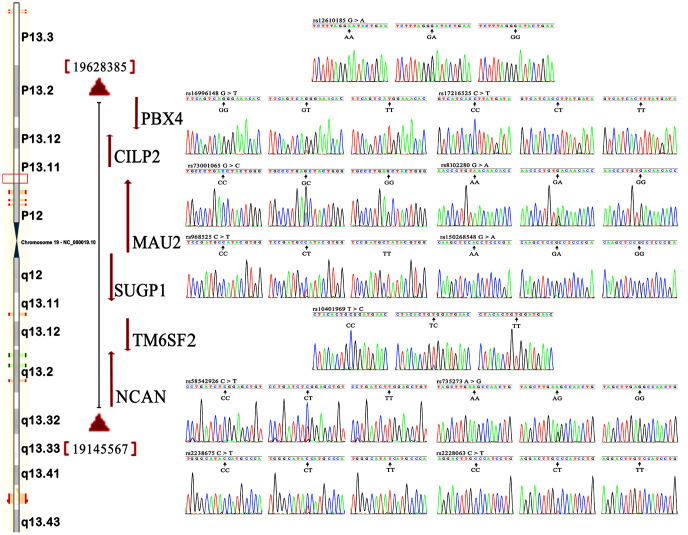
**Locations and partial nucleotide sequences of the *NCAN*, *TM6SF2*, *CILP2*, *PBX4*, *SUGP1* and *MAU2* SNPs.** *NCAN*, the neurocan gene; *TM6SF2*, the transmembrane 6 superfamily member 2 gene; *CILP2*, the cartilage intermediate layer protein 2 gene; *PBX4*, the PBX homeobox 4 gene; *SUGP1*, the SURP and G-patch domain containing 1 gene; *MAU2*, the *MAU2* sister chromatid cohesion factor gene.

Figure 1 shows the locations, as well as the partial nucleotide sequences, of the *NCAN*, *TM6SF2*, *CILP2*, *PBX4*, *SUGP1* and *MAU2* SNPs, which are located on chromosome 19. The genotypes of 12 SNPs were confirmed by direct sequencing. As mentioned in Table 2, the genotypic distribution of 12 SNPs substantially conformed to Hardy-Weinberg equilibrium (HWE) in the hyperlipidemia and normal. The genotypic and allelic frequencies of 12 SNPs in the *NCAN*, *TM6SF2*, *CILP2*, *PBX4*, *SUGP1* and *MAU2* were substantially different between the hyperlipidemia and normal groups (Tables 2 and 3). The allelic frequencies of rs2238675C, rs2228603T, rs58542926T, rs735273G, rs16996148T, rs17216525T, rs12610185A, rs1040 1969T, rs73001065G, rs8102280G, rs150268548A, and rs968525T were substantially greater in hyperlipidemic individuals than in normal subjects (*P* < 0.05-*P* < 0.001, for all).

**Table 2:** Comparison of the genotype frequencies between the normal and hyperlipidemia groups [n (%)].

**SNP**	**Genotype**	**Normal (n=1248)**	**Hyperlipidemia(n=1248)**	**χ^2^**	***P***
*NCAN* rs2238675 C>T	CC	135(10.8)	164(13.1)	6.395	0.041
	CT	551(44.2)	578(46.3)		
	TT	562(45.0)	506(40.5)		
	*P*_HWE_	0.891	0.245		
*NCAN* rs2228603 C>T	CC	454(36.4)	375(30.1)	15.334	3E-006
	CT	597(47.8)	618(49.5)		
	TT	197(15.8)	255(20.4)		
	*P*_HWE_	0.748	0.071		
*TM6SF2* rs58542926 C>T	CC	713(57.1)	663(53.1)	6.542	0.038
	CT	461(36.9)	484(38. 8)		
	TT	74(5.9)	101(8.1)		
	*P*_HWE_	0.941	0.065		
*TM6SF2* rs735273 A>G	AA	989 (79.3)	923(74.0)	10.317	0.006
	AG	244 (19.6)	301(24.1)		
	GG	15(1.2)	24(1.9)		
	*P*_HWE_	0.887	0.238		
*CILP2* rs16996148 G>T	GG	984 (78.9)	667 (53.5)	187.550	E-015
	GT	243 (19.5)	491(39.3)		
	TT	21 (1.7)	90(7.2)		
	*P*_HWE_	0.891	0.245		
*CILP2* rs17216525 C>T	CC	64(5.1)	36(2.9)	26.428	3E-8
	CT	437(35.0)	348(27.9)		
	TT	747(59.9)	864(69.2)		
	*P*_HWE_	0.778	0.651		
*PBX4* rs12610185 G>A	GG	241(19.3)	176(14.1)	21.127	E-7
	GA	614(49.2)	584(46.8)		
	AA	393(31.5)	488(39.1)		
	*P*_HWE_	0.886	0.628		
*SUGP1* rs10401969 T>C	TT	437(35.0)	476(38.1)	7.034	0.030
	TC	608(48.7)	613(49.1)		
	CC	203(16.3)	159(12.7)		
	*P*_HWE_	0.781	0.104		
*MAU2* rs73001065 G>C	GG	63(5.05)	81(6.5)	14.640	0.001
	GC	435(34.9)	511(41.0)		
	CC	750(60.1)	656(52.6)		
	*P*_HWE_	0.884	0.111		
*MAU2* rs8102280 G>A	GG	582(46.4)	640(51.3)	6.546	0.038
	GA	540(43.3)	507(40.6)		
	AA	126(10.3)	101(8.1)		
	*P*_HWE_	0.872	0.566		
*MAU2* rs150268548 G>A	GG	227 (18.20)	171(13.7)	16.568	7E-006
	GA	611(49.0)	582(46.6)		
	AA	410 (32.9)	495(39.7)		
	*P*_HWE_	0.664	0.064		
*MAU2* rs968525 C>T	CC	228 (18.3)	185(14.8)	6.058	0.048
	CT	576(46.2)	581(46.6)		
	TT	444(35.6)	482(38.6)		
	*P*_HWE_	0.521	0.073

*NCAN*: the neurocan gene. *TM6SF2*: the transmembrane 6 superfamily member 2 gene. *CILP2*: the cartilage intermediate layer protein 2 gene. *PBX4*: the PBX homeobox 4 gene. *SUGP1*: the SURP and G-patch domain containing 1 gene. *MAU2*: the MAU2 sister chromatid cohesion factor gene. HWE: Hardy-Weinberg equilibrium.

**Table 3:** Comparison of the allele frequencies between the normal and hyperlipidemia groups [n (%)].

**SNP**	**Allele**	**Normal (n=1248)**	**Hyperlipidmia (n=1248)**	**χ^2^**	***P***
*NCAN* rs2238675	C/T	821(33.0)/1675(67.1)	906(36.3)/1590(63.7)	6.396	0.011
*NCAN* rs2228603	C/T	1505(60.3)/991(39.7)	1368(54.8)/1128(45.2)	15.390	E-005
*TM6SF2* rs58542926	C/T	1887(75.6)/609(24.4)	1810(72.5)/686(27.5)	6.182	0.013
*TM6SF2* rs735273	A/G	2222(89.0)/274(11.0)	2147(86.0)/349(14.0)	10.316	0.001
*CILP2* rs16996148	G/T	2211 (88.6)/285(11.4)	1825(73.1)/671(26.9)	192.770	4E-012
*CILP2* rs17216525	C/T	565(22.6)/1931(77.4)	420(16.8)/2076(83.2)	26.592	3E-007
*PBX4* rs12610185	G/A	1096(43.9)/1400(56.1)	936(37.5)/1560(62.5)	21.247	2E-007
*SUGP1* rs10401969	T/C	1482(59.4)/1014(40.6)	1565(62.7)/931(37.3)	5.803	0.016
*MAU2* rs73001065	G/C	561(22.5)/1935(77.5)	673(27.0)/1823(73.0)	13.503	9E-004
*MAU2* rs8102280	G/A	1704(68.3)/792(31.7)	1787(71.6)/709(28.4)	6.563	0.010
*MAU2* rs150268548	G/A	1065(42.7)/1431(57.3)	924(37.0)/1572(63.0)	16.616	6E-005
*MAU2* rs968525	C/T	1031(41.3)/1465(58.7)	951(38.1)/1545(61.9)	5.355	0.021

*NCAN*: the neurocan gene. *TM6SF2*: the transmembrane 6 superfamily member 2 gene. *CILP2*: the cartilage intermediate layer protein 2 gene. *PBX4*: the PBX homeobox 4 gene. *SUGP1*: the SURP and G-patch domain containing 1 gene. *MAU2*: the MAU2 sister chromatid cohesion factor gene.

### Genotypes and serum lipid profiles

**Figure 2:**
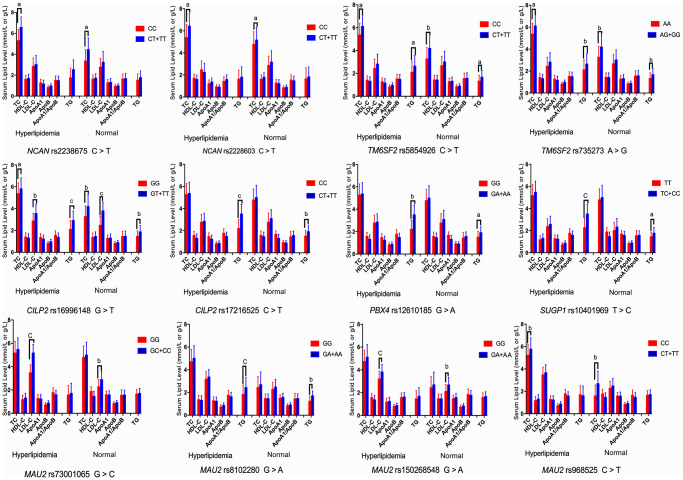
**Association of the *NCAN*, *TM6SF2*, *CILP2*, *PBX4*, *SUGP1* and *MAU2* genotypes and serum lipid parameters in the normal and hyperlipidemia groups.** TC, total cholesterol; HDL-C, high-density lipoprotein cholesterol; LDL-C, low-density lipoprotein cholesterol; ApoA1, apolipoprotein A1; ApoB, apolipoprotein B; TG, triglyceride. ^a^*P* < 0.004, ^b^*P* < 0.001, and ^c^*P* < 0.0001.

The associations among the genotypes of the *NCAN*, *TM6SF2*, *CILP2*, *PBX4*, *SUGP1* and *MAU2* SNPs and serum lipid concentrations are presented in Figure 2. The minor allele carriers had higher serum levels of TC (*NCAN* rs2238675, *NCAN* rs2228603, *TM6SF2* rs58542926, *TM6SF2* rs735273, *CILP2* rs16996148, and *MAU2* rs968525), TG (*TM6SF2* rs58542926, *TM6SF2* rs735273, *CILP2* rs16996148, *CILP2* rs17216525, *PBX4* rs12610185, *SUGP1* rs10401969, and *MAU2* rs8102280), and LDL-C (*CILP2* rs16996148, *MAU2* rs73001065, and *MAU2* rs150268548) than the minor allele noncarriers in both hyperlipidemia and normal groups (*P* < 0.004 for all).

### Haplotype-based association with hyperlipidemia

**Figure 3:**
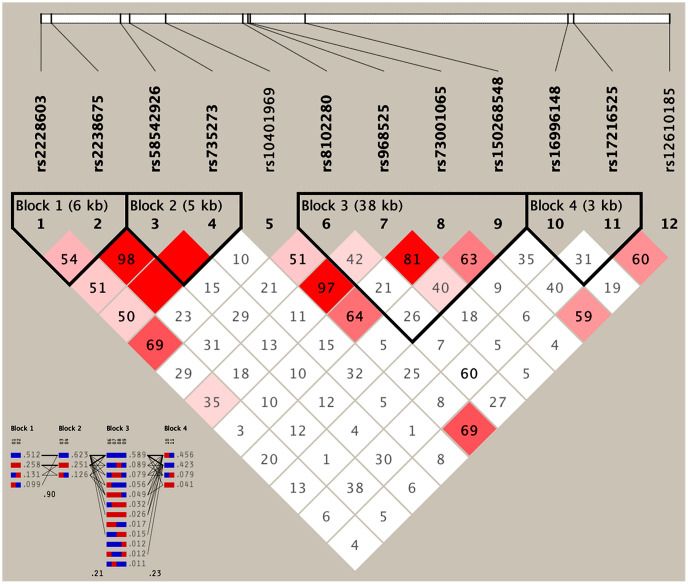
**Results of linkage disequilibrium (LD) analyses of the *NCAN*, *TM6SF2*, *CILP2*, *PBX4*, *SUGP1* and *MAU2* SNPs.**

**Figure 4:**
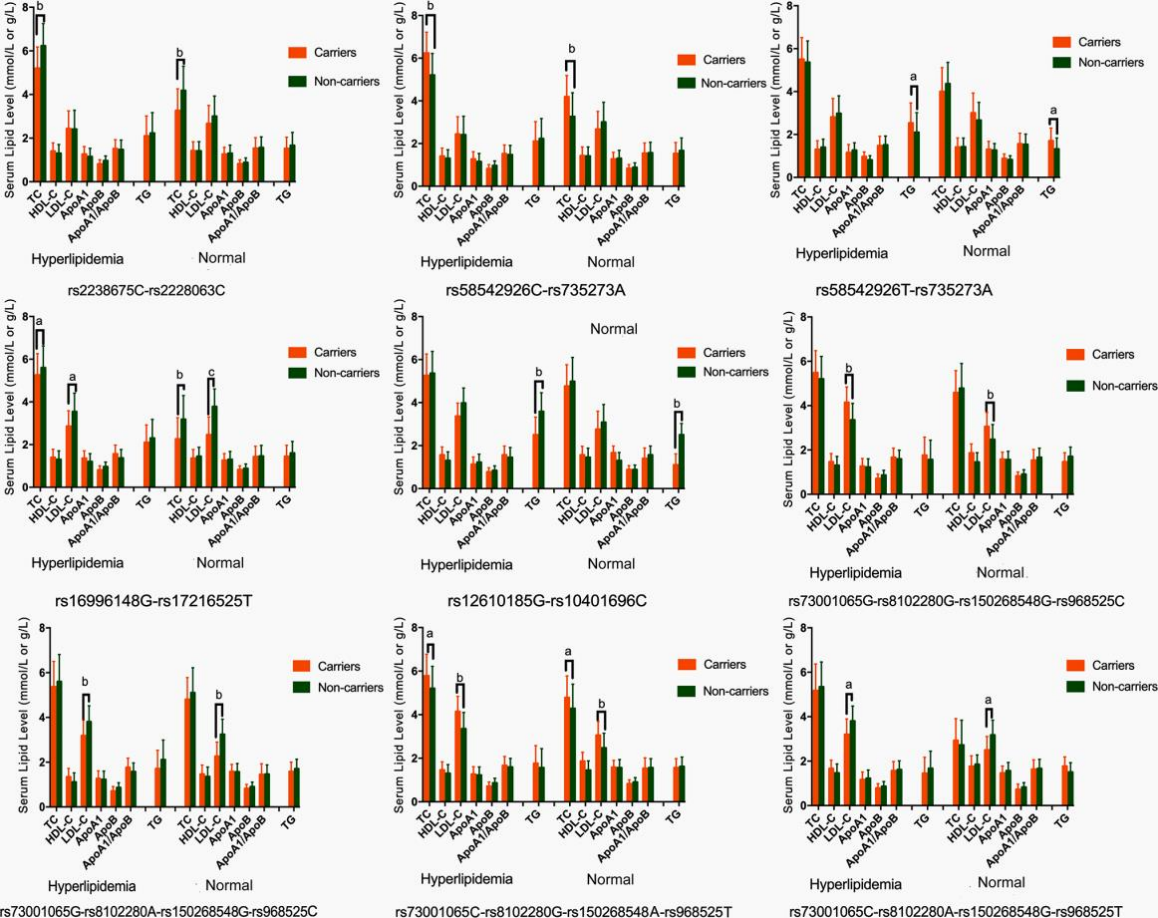
**Association of the *NCAN*, *TM6SF2*, *CILP2*, *PBX4*, *SUGP1* and *MAU2* haplotypes and serum lipid parameters in the normal and hyperlipidemia groups.** TC, total cholesterol; HDL-C, high-density lipoprotein cholesterol; LDL-C, low-density lipoprotein cholesterol; ApoA1, apolipoprotein A1; ApoB, apolipoprotein B; TG, triglyceride. ^a^*P* < 0.006, ^b^*P* < 0.001. and ^c^*P* < 0.0001.

As presented in Table 4, the most common haplotypes were the *NCAN* C-T, *TM6SF2* T-A, *PBX4-SUGP1* G-T and *MAU2* C-G-A-T (≥ 30%, in all samples). The incidences of the *NCAN* C-C (G2), *TM6SF2* T-A (G3), *TM6SF2* C-A (G5), *CILP2* G-T (G6), *PBX4-SUGP1* G-C (G8), *MAU2* G-G-G-C (G9), *MAU2* G-A-G-C (G10), *MAU2* C-G-A-T (G12), and *MAU2* C-A-G-T (G13) haplotypes were significantly different between the hyperlipidemia and normal groups (*P* < 0.05 for all). In addition, the haplotypes of G2, G6, G8, and G13 showed a protective effect, whereas all of the G3, G5, G9 and G12 haplotypes showed an inverse effect (*P* < 0.05-0.001, respectively). The detected sites that were elucidated by multiple locus LD were not fully statistically independent in the participants. As presented in Figure 3, both the LD and the haplotypes block the combination of two groups. Figure 4 shows that carriers with the detected gene-gene interaction haplotypes had higher serum TC (rs58542926C-rs735273A and rs73001065C-rs8102280G-rs150268548A-rs968525T), LDL (rs73001065G-rs8102280G-rs150268548G-rs968525C, and rs73001065C-rs8102280G-rs150268548A-rs968525T), and TG (rs58542926T-rs735273A) levels than the haplotype non-carriers. .

**Table 4:** Comparison of the haplotype frequencies between the normal and hyperlipidemia groups [n (frequency)].

**NoO.**	**Haplotype**	**Hyperlipidemia**	**Normal**	**χ^2^**	***P***	**OR (95% CI)**
G1	*NCAN* C-T	815.80(0.3)	818.97(0.3)	0.091	0.762	1.0 (0.9-1.2)
G2	*NCAN* C-C	0.08(0.0)	0.20(0.0)	20.637	6E-006	0.7 (0.7-0.8)
G3	*TM6SF2* T-A	626.97(0.3)	611.80(0.3)	16.728	4E-005	1.4 (1.2-1.6)
G4	*TM6SF2* C-G	20.95(0.0)	0.00(0.0)	0.446	0.504	1.1 (0.9-1.2)
G5	*TM6SF2* C-A	84.00(0.0)	211.15(0.1)	10.752	0.001	1.3 (1.1-1.5)
G6	*CILP2* G-T	0.00(0.0)	12.00(0.0)	14.534	E-004	0.7 (0.6-0.8)
G7	*PBX4-SUGP1* G-T	1524.00(0.6)	1523.83(0.6)	0.049	0.826	1.0 (0.9-1.1)
G8	*PBX4-SUGP1* G-C	35.96(0.0)	36.16(0.0)	12.128	5E-004	0.8 (0.7-0.9)
G9	*MAU2* G-G-G-C	480.04(0.2)	371.85(0.2)	15.976	6E-005	1.4 (1.2-1.6)
G10	*MAU2* G-A-G-C	61.44(0.3)	86.22(0.0)	3.662	0.036	0.7 (0.5-1.0)
G11	*MAU2* C-A-A-T	203.71(0.0)	252.01(0.1)	4.376	0.557	0.8 (0.7-0.9)
G12	*MAU2* C-G-A-T	1461.50(0.6)	1341.77(0.5)	20.71	5E-006	1.3 (1.2-1.5)
G13	*MAU2* C-A-G-T	645.79(0.3)	753.77(0.3)	8.189	0.004	0.8 (0.7-0.9)

*NCAN*: the neurocan gene. *TM6SF2*: the transmembrane 6 superfamily member 2 gene. *CILP2*: the cartilage intermediate layer protein 2 gene. *PBX4*: the PBX homeobox 4 gene. *SUGP1*: the SURP and G-patch domain containing 1 gene. *MAU2*: the MAU2 sister chromatid cohesion factor gene. The haplotype is combined with *NCAN* rs2238675-rs2228603, *TM6SF2* rs58542926-rs735273, *CILP2* rs16996148-rs17216525, *PBX4-SUGP1* rs12610185-rs10401969, and *MAU2* rs73001065-rs8102280-rs150268548-rs968525.

### Gene-gene (G × G) interaction-based association with hyperlipidemia

**Figure 5:**
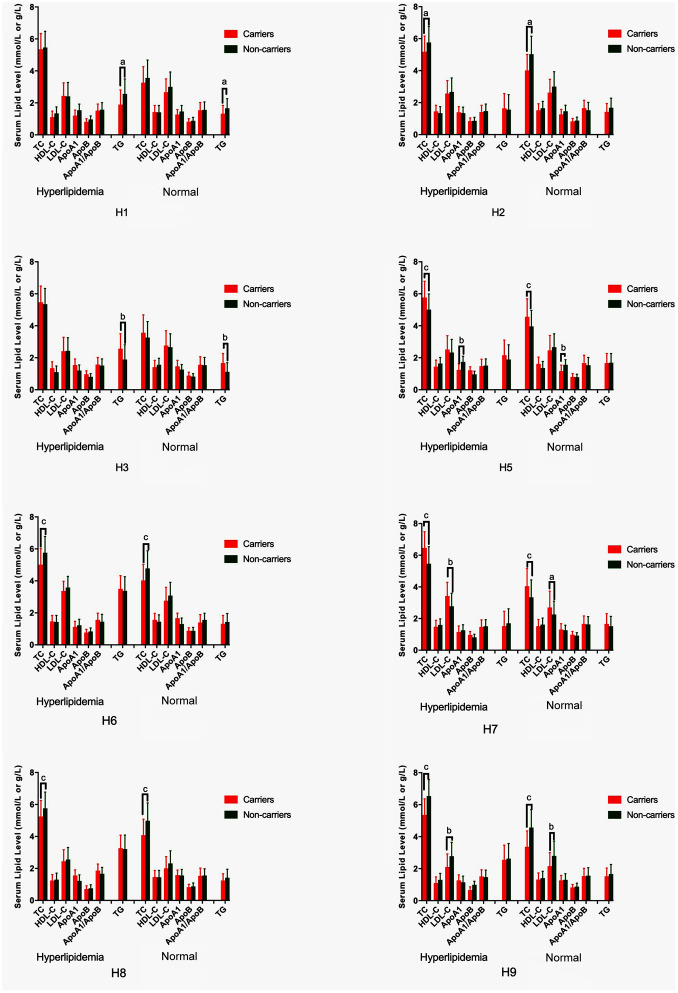
**G × G haplotype-based association with serum lipid levels in normal and hyperlipidemic individuals.** TC, total cholesterol; HDL-C, high-density lipoprotein cholesterol; LDL-C, low-density lipoprotein cholesterol; ApoA1, apolipoprotein A1; ApoB, apolipoprotein B; TG, triglyceride. ^a^*P* < 0.006, ^b^*P* < 0.001, and ^c^*P* < 0.0001.

As shown in Table 5, the most common G × G interaction was C-C-C-A-G-C-A-T-C-G-A-T (H1, > 15%, in all samples). The frequencies of the C-C-C-A-G-C-A-T-C-G-A-T (H1), T-C-C-A-G-C-A-T-C-G-A-T (H2), T-T-C-A-G-C-A-T-C-G-A-T (H3), C-C-C-A-G-C-A-T-C-A-G-C (H5), C-C-C-A-G-C-G-C-G-A-G-C (H6), T-T-T-A-G-C-G-C-C-G-A-T (H7), T-T-T-A-G-C-G-C-C-A-G-C (H8), and T-T-T-A-G-C-A-T-C-A-G-C (H9) G × G interactions were significantly different between the normal and hyperlipidemia groups (*P* < 0.05 for all). Meanwhile, the G × G interactions of H1, H2, H6, H8 and H9 contributed to a protective effect, while the G × G interaction of H3, H5 and H7 showed an inverse effect. The H2, H6, H8 and H9 carriers had low TC levels, but the H5 and H7 carriers had high TC levels; the H1 carriers had low TG levels, but the H3 carriers had high TG levels; the H7 carriers had high LDL-C levels, and the H9 carriers had low LDL-C levels; and the H5 carriers had high ApoA1 levels in both the normal and hyperlipidemia groups (Figure 5; *P* < 0.006 for all).

**Table 5:** Comparison of G × G interaction frequencies between the normal and hyperlipidemia groups [n (frequency)].

**No**	**G × G inteaction**												**Hyperlipidemia**	**Normal**	***x*^2^**	***P***	**OR (95%CI)**
	A	B	C	D	E	F	G	H	I	J	K	L					
H1	C	C	C	A	G	C	A	T	C	G	A	T	440.78(0.177)	422.58(0.169)	1.961	0.021	0.9 (0.8-1.1)
H2	T	C	C	A	G	C	A	T	C	G	A	T	219.96(0.088)	251.06(0.101)	9.170	0.002	0.6 (0.6-0.9)
H3	T	T	C	A	G	C	A	T	C	G	A	T	162.55(0.065)	99.41(0.040)	9.610	0.002	1.5 (1.2-2.0)
H4	T	T	T	A	G	C	A	T	C	G	A	T	119.10(0.048)	91.43(0.037)	1.178	0.278	1.2 (0.9-1.6)
H5	C	C	C	A	G	C	A	T	C	A	G	C	109.61(0.044)	46.95(0.019)	19.385	E-005	2.2 (0.9-1.6)
H6	C	C	C	A	G	C	G	C	G	A	G	C	55.83(0.022)	87.32(0.035)	11.910	6E-004	0.6 (1.5-3.0)
H7	T	T	T	A	G	C	G	C	C	G	A	T	95.07(0.038)	33.78(0.014)	23.537	E-006	2.6 (1.8-3.9)
H8	T	T	T	A	G	C	G	C	C	A	G	C	42.20(0.017)	76.13(0.031)	14.956	E-004	0.5 (0.3-0.7)
H9	T	T	T	A	G	C	A	T	C	A	G	C	16.45(0.007)	100.45(0.040)	74.461	7E-018	0.1 (0.1-0.2)

**A**: *NCAN* rs2238675 C>T. **B**: *NCAN* rs2228603 C>T. **C**: *TM6SF2* rs58542926 C>T. **D**: *TM6SF2* rs735273 A>G. **E**: *CILP2* rs16996148 G>T. **F**: *CILP2* rs17216525 C>T. **G**: *PBX4* rs12610185 G>A. **H**: *SUGP1* rs10401969 T>C. **I**: *MAU2* rs73001065 G>C. **J**: *MAU2* rs8102280 G>A. **K**: *MAU2* rs150268548 G>A. **L**: *MAU2* rs968525 C>T. *NCAN*: the neurocan gene. *TM6SF2*: the transmembrane 6 superfamily member 2 gene. *CILP2*: the cartilage intermediate layer protein 2 gene. *PBX4*: the PBX homeobox 4 gene. *SUGP1*: the SURP and G-patch domain containing 1 gene. *MAU2*: the MAU2 sister chromatid cohesion factor gene.

### G × G and gene-environment (G × E) interactions on hyperlipidemia

**Figure 6:**
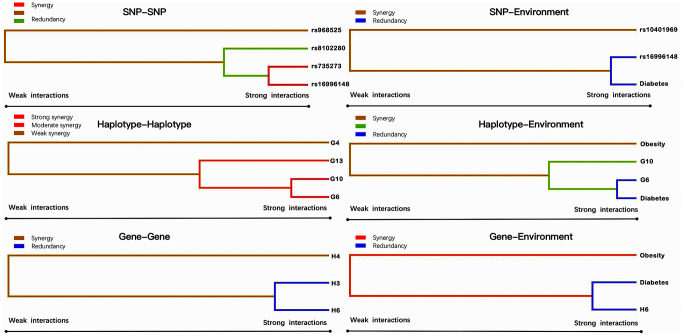
**Various sorts of gene-gene and gene-environment interaction dendrograms.** Elements with strong interactions appear close together, and elements with weak interactions appear distant from each other.

**Figure 7:**
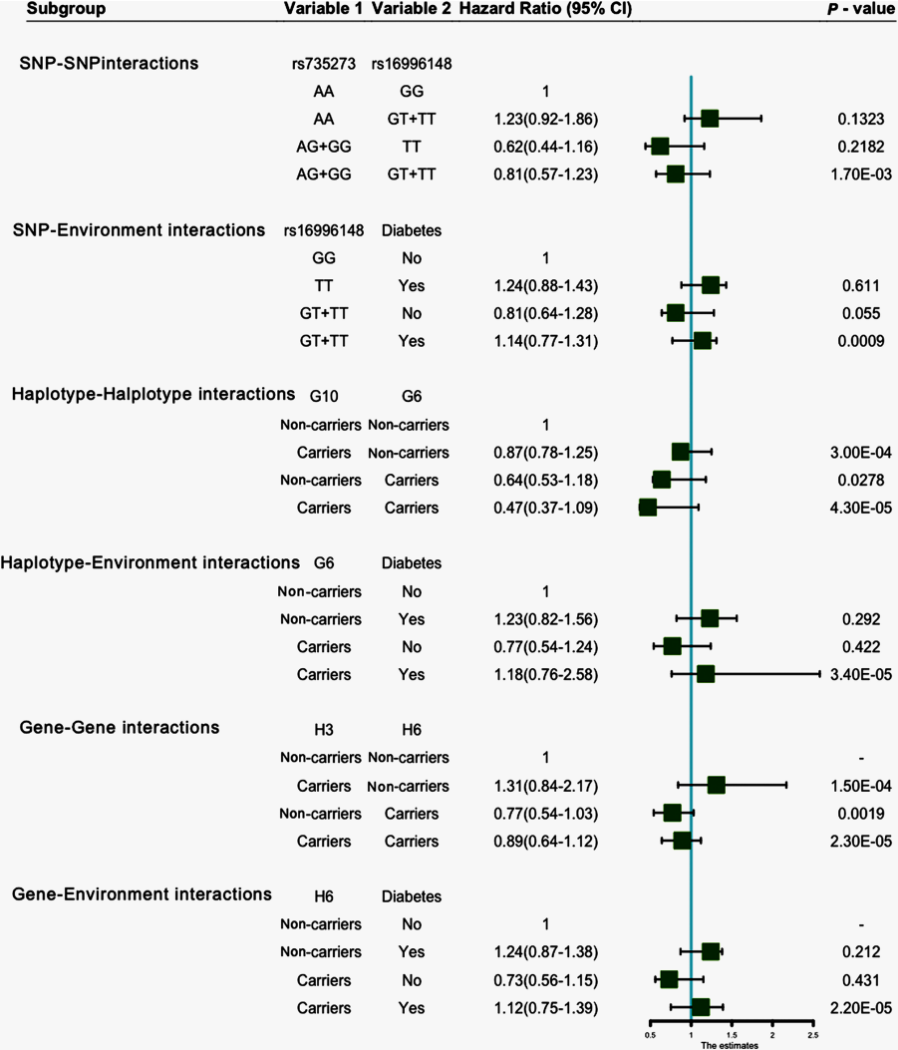
**SNP-SNP, SNP-environment, haplotype-haplotype, haplotype-environment, gene-gene and gene-environment interactions on the risk of hyperlipidemia.**

Entropy-based interaction dendrogram (Figure 6) and proportional hazard model results (Figure 7) show the strongest synergy of SNP-SNP interaction between rs735273 and rs16996148 and haplotype-haplotype interaction between G10 and G6. However, these results showed a redundancy effect in SNP-environment interaction (rs16996148 and diabetes), haplotype-environment interaction (G6 and diabetes), gene-gene interaction (H3 and H6) and gene-environment interaction (H6 and diabetes). We also established that the rs735273 AA and rs16996148 GT/GG genotypes increased the risk of hyperlipidemia, whereas the rs735273 AG/GG, rs16996148 TT, rs735273 AG/GG and rs16996148 GT/TT genotypes decreased the risk of hyperlipidemia. SNP-environment interaction and rs16996148 and diabetes indicated that the rs16996148 SNP decreased the risk of hyperlipidemia, whereas rs16996148 GT/TT and diabetes, rs16996148 TT and diabetes increased the risk of hyperlipidemia. The haplotype-haplotype interaction showed that G10 (*MAU2* G-A-G-C) and G6 (*CILP2* G-T) carriers could reduce the risk of hyperlipidemia compared with G10 or G6 carriers. With regard to the gene-gene interaction between H3 (T-T-C-A-G-C-A-T-C-G-A-T) and H6 (C-C-C-A-G-C-G-C-G-A-G-C) carriers, we found that the latter showed an inferior risk of hyperlipidemia, while the former indicated an augmented probability of hyperlipidemia. As a genotype-environment interaction was considered, G6 (*CILP2* G-T) carriers and diabetes increased the risk of hyperlipidemia. A similar result was shown in the gene-environment interaction between H6 (C-C-C-A-G-C-G-C-G-A-G-C) carriers and diabetes.

## DISCUSSION

The major new findings in this study were as follows: (1) The study showed the single nucleotide mutation frequencies, haplotype frequencies and interaction of G × G interlocus frequencies among 12 *NCAN, TM6SF2, CILP2, PBX4, SUGP1* and *MAU2* SNPs in the people from Southwest China for the first time; (2) It also presented new evidence that single nucleotide mutation, haplotype, G × G and G × E interactions among the *NCAN, TM6SF2, CILP2, PBX4, SUGP1* and *MAU2* SNPs are probably closely associated with serum lipid levels; (3) We established some new diversity effects from the interactions of SNP-SNP, SNP-environment, haplotype-haplotype, haplotype-environment, G × G and G × E; and (4) We also found different interactions that augmented the risk of hyperlipidemia.

Hyperlipidemia is the main risk factor that can result in CVD, which accounts for approximately 4 million deaths each year worldwide [25, 26]. High levels of TC can contribute to the risk for CAD [27], ischemic cerebrovascular accident [28], aortic dissection and peripheral arterial disease [29]. It has been demonstrated that TG levels have an intense association with non-alcoholic fatty liver disease (NAFLD) and metabolic syndrome [30]. NAFLD and metabolic syndrome have also been reported to be independent of the risk factors for subclinical atherosclerosis [31, 32]. Previous studies have demonstrated that serum lipid levels could be affected by multiple environmental factors, such as unhealthy diet [33], lifestyle (e.g., smoking, excessive alcohol consumption, insufficient exercise) [34–36], genetic factors [37], and their interactions [38].

This study identified that the variants of *NCAN, TM6SF2, CILP2, PBX4, SUGP1* and *MAU2* were related to serum lipid concentrations. Moreover, there were substantial differences in the genotypic and allelic frequencies of 12 SNPs between the normal and hyperlipidemia groups. These outcomes suggest that genetic factors are associated with the prevalence of hyperlipidemia. When we analyzed the relationship between SNPs and hyperlipidemia, the rs735273 and rs16996148 SNPs were found to decrease the risk of hyperlipidemia. However, the interaction of the SNP-environment showed that subjects with the rs16996148 SNP and diabetes had an increased risk of hyperlipidemia. We also found similar results in the interactions of haplotype-environment, G × G and G × E. A plausible interpretation for these findings is that metabolic disorder might occur due to the combined influence of people’s behavior, environmental and genetic factors [39, 40]. More than 50% of the diet of southern Chinese populations includes cereals [41], which significantly lack some important micronutrients, such as vitamins and dietary fiber. These populations prefer rice, refreshing sour, spicy and sweet food. Furthermore, these populations all prefer food containing many saturated fatty acids, such as pork, beef and animal organ offal [42]. Long-chain dietary saturated fatty acids have shown detrimental consequences on lipid metabolism in blood, especially resulting in higher levels of plasma TC and TG [43, 44].

Unhealthy lifestyles (e.g., unhealthy diet, smoking, excessive alcohol intake and lack of exercise) have been closely connected with abnormal serum lipid levels [45]. Compared with the normal groups, there was a higher percentage of smoking and alcohol intake in the hyperlipidemia group. A large number of Southwest Chinese adults enjoy drinking. Most people who live in rural areas usually make wine themselves by using corns, cereals and cassava. It has been documented that alcohol could elevate serum levels of HDL-C and benefit CAD [46, 47]. However, it has also been reported that the elevation of HDL-C levels was set off by increased smoking levels. Smoking could increase the serum concentrations of TC, TG and LDL-C, but it could decrease serum levels of HDL-C [48, 49]. This phenomenon may be a suitable explanation for the current results of serum lipid levels between the two groups. There might be an effect of modifiable or nonmodifiable risk factors on genetic variants identified in GWAS of disease. Recently, a number of variants have been identified to be connected with lifestyle behaviors and health outcomes in GWAS. From the example of tobacco and alcohol research that we discussed above, behavioral phenotypes can be predicted by a genetic variant, which has been shown in GWAS of disorders that informally interact with these activities. It is important to explain GWAS findings [50].

Dyslipidemia is the result of a combination of genetic and environmental factors that have been universally recognized worldwide [51, 52]. China is a multiethnic country with 56 ethnic groups [53]. Han nationality is the largest ethnic group, and the rest of 55 ethnic groups are distributed in different areas of the country. The genotypic and allelic frequencies of many SNPs in some genes were inconsistent in diverse racial/ethnic groups [54–56]. There may also be an ethnic difference in lifestyle and environmental factors, as well as in genetic background. To the best of our knowledge, the *TM6SF2* rs58542926 SNP increased the risk of NAFLD in the eastern Chinese Han population [57]. The SNP of rs16996148 in *NCAN-CILP2 or NCAN/CILP2/PBX4* was significantly associated with dyslipidemia in the midlands and east of the Chinese Han population [8, 58]. The studies mentioned above suggested that genetic variants of those genes in chromosome 19p13 confer susceptibility to dyslipidemia in the Chinese populations. However, the relationship between dyslipidemia and *SUGP1* and *MAU2* is not clear in the Chinese populations, and the association between SNPs, gene-gene, and gene-environment interactions and dyslipidemia is still limited. With the rapid development of biomedicine technology, we are entering a precision medicine era, and precision medicine seeks to identify and classify individual patients such that optimal treatment decisions can be made. It is essential to explore the *NCAN-TM6SF2-CILP2-PBX4-SUGP1-MAU2* SNPs, gene-gene and gene-environment interactions on serum lipid levels in Southeast China and other areas of Chinese populations. These results may help us to take precise treatment for dyslipidemia and decrease the risk of CVD.

The current study has several limitations. First, the sample size is comparatively small. Thus, additional studies with large sample sizes are necessary. Second, lower numbers of individuals are obtainable for minor allele frequency (MAF) of certain variants, and it is relatively weak in calculating a strong power. Third, numerous unmeasured environmental and genetic factors must be determined, such as dietary patterns, physical exercises, and energy intake. Finally, we should define the relevance of this finding with a high criterion in further studies, including incorporating the genetic information of the *NCAN, TM6SF2, CILP2, PBX4, SUGP1* and *MAU2* single nucleotide mutations, haplotypes, interactions of G × G and G × E from *in vivo* to *in vitro*, and testing the effect of genetic variants with different molecular biological levels, such as genetic transcription and expression.

In conclusion, this study shows potential interactions among the *NCAN, TM6SF2, CILP2, PBX4, SUGP1* and *MAU2*, environment and serum lipid levels in hyperlipidemia subjects. Our findings also showed that the interactions increased the risk of hyperlipidemia over single-locus tests. In addition, these factors exhibit distinctive collaboration or redundancy effects on morbidity.

## MATERIALS AND METHODS

### SNP selection

Twelve SNPs in the *NCAN, TM6SF2, CILP2, PBX4, SUGP1* and *MAU2* were selected as follows: (1) *NCAN*, which was associated with serum lipid levels, was selected from a previous GWAS. The gene clusters of *TM6SF2*-*CILP2*-*PBX4*-*SUGP1*-*MAU2* were closely associated with lipid metabolism and *NCAN*. (2) Information regarding Tagging SNPs, functional SNPs, and predicted SNPs can be found in our previous article [23]. (3) SNP information was obtained from NCBI dbSNP Build 132 (http://www.ncbi.nlm.nih.gov/SNP/), which can be found in Supplementary Table 1. The MAF was restricted to greater than 1% in SNPs. (4) There might be some association between those SNPs and serum lipid levels or cardio cerebral vascular diseases in previous studies. (5) *NCAN* rs2238675-rs2228603, *TM6SF2* rs58542926-rs735273, *CILP2* rs16996148-rs17216525, *PBX4-SUGP1* rs12610185-rs10401969, and *MAU2* rs73001065-rs8102280-rs150268548-rs968525 were chosen by the block-based method. This strategy is facilitated by the associations among tagging SNPs and is demonstrated as LD (*D*′ > 0.7).

### Subjects

The sample sizes were calculated by Quanto software (Version 1.2, http://biostats.usc.edu/Quanto.html) at the beginning of this study, and they were sufficient to satisfy the statistical power. A total of 1248 unrelated patients with hyperlipidemia were enrolled from the First Affiliated Hospital, Guangxi Medical University from Sep. 1, 2016 to Dec. 31, 2018. Participants were 18 to 80 years old (mean 55.98 ± 12.78 years), and patients with a family history of hyperlipidemia were excluded. Meanwhile, a total of 1248 randomly selected adults served as the control group. They underwent periodical medical check-ups, and their age, gender and ethnic group were matched to the patients. They were 18 to 80 years old (mean 56.87 ± 12.12 years). There was no history of major diseases in any participants. None of the participants took any medications that might have any impact on lipid metabolism. This study design was approved by the Ethics Committee of the First Affiliated Hospital, Guangxi Medical University (No. Lunshen 2014-KY-Guoji-001; Mar. 7, 2014). Informed consent was obtained from all participants.

### Clinical data

The clinical data were obtained by means of a universally standardized technique [38]. Standardized questionnaires were administered to acquire details of demographics, socioeconomic standing and lifestyle dynamics. The status of cigarette smoking was categorized into ≤ 20 cigarettes per day and > 20 cigarettes per day [59]. Alcohol intake was classified based on the grams of alcohol per day: ≤ 25 and > 25 [23]. Details regarding other factors, such as height, weight, waist circumference, blood pressure, and BMI (kg/m^2^), were also acquired.

### Biochemical measurements

Venous blood samples were acquired following 12 h of fasting. TC, HDL-C, LDL-C and TG concentrations in serum were detected by means of Tcho-1, TG-LH (RANDOX Laboratories, UK), Cholestest N HDL, and Cholestest LDL (Daiichi Pure Chemicals Co., Ltd., Japan) kits, respectively. ApoA1 and ApoB concentrations in serum were determined by immunoassay (RANDOX Laboratories). Detection of all samples was completed with an autoanalyzer (Hitachi Ltd., Japan) [60].

### Diagnostic criteria

The standard values of serum lipid levels in our clinical biochemistry laboratory were as follows: TC (3.10–5.17 mmol/L), TG (0.56–1.70 mmol/L), HDL-C (1.16–1.42 mmol/L), LDL-C (2.70–3.10 mmol/L), ApoA1 (1.20–1.60 g/L), ApoB (0.80–1.05 g/L) and the ApoA1/ApoB ratio (1.00–2.50). Hyperlipidemia was diagnosed with serum levels of TC > 5.17 mmol/L and/or TG > 1.70 mmol/L [61, 62]. The diagnosis of hypertension was made as per the Seventh Report of Joint National Committee (JNC-7) [63]. BMI was classified as normal (< 24 kg/m^2^), overweight (24–28 kg/m^2^) or obese (> 28 kg/m^2^).

### Genotyping

Extraction of genomic DNA was accomplished by utilizing the conventional phenol-chloroform method in venous blood leucocytes. Genotyping of the 12 variants was performed on the Snapshot of next generation sequencing technology platform HiSeq XTen (Illumina, USA) in Sangon Biotech Co., Ltd. (Shanghai, China). The details regarding sense and antisense primers are provided in Supplementary Table 2.

### Statistical analyses

SPSS 22.0 (IBM SPSS Inc., USA) was employed to analyze the data. Quantitative variables of normally distributed data are represented as the mean ± SD, while serum TG levels of non-normally distributed data are represented as medians and interquartile ranges. Typical features between the normal and hyperlipidemia groups were compared by means of analysis of covariance. Distribution of the genotypes and interactions of alleles, haplotypes, G × G between normal and hyperlipidemia groups were examined by chi-square test; the HWE, pairwise LD, haplotype frequencies and G × G interaction containing the variants were computed by means of Haploview (version 4.2; Broad Institute of MIT and Harvard). The pattern of pairwise LD among 12 SNPs was tested by *D*′ using Haploview software. We employed Univariant to test associations between genotypes, haplotypes, G × G interactions and lipid phenotypic variations. *P* < 0.004 represented statistical significance in the association between any variants and lipid phenotypic variations (equivalent to *P* < 0.05 after adjusting for 12 independent tests by the Bonferroni correction). The association between genotypes, alleles, haplotypes, G × G interactions and lipid phenotypic variants was performed using unconditional logistic regression evaluation. Other parameters were adjusted for the data analysis. The greatest interaction pattern among genes, SNPs and environmental exposures was screened by means of generalized multifactor dimensionality reduction [64]. The cross-validation consistency score was performed to identify the best model of selected interaction among all probabilities. The testing balanced accuracy was a measure of the degree to which the interaction precisely calculates case-control status with scores between 0.50 (representing that the model projects no better than chance) and 1.00 (representing impeccable prediction). Finally, to evaluate whether an identified model is significant, we used a sign test or a permutation test for accuracy of prediction.

### Availability of data and materials

The datasets generated during the present study are not publicly available, because detailed genetic information of each participant was included in these materials.

## Supplementary Material

Supplementary Tables
