# Supplementary material for: Association of the NCAN-TM6SF2-CILP2-PBX4-SUGP1-MAU2 SNPs and gene-gene and gene-environment interactions with serum lipid levels
Source: Aging (Albany NY). 2020 Jun 22;12(12):11893–913. doi: 10.18632/aging.103361 (PMC7343441; doi:10.18632/aging.103361)
Supplement: Supplementary Tables [file aging-12-103361-s001..pdf]

SUPPLEMENTARY TABLES

Supplementary Table 1. Characteristics of the 19p13.11 gene mutations.

SNV ID (rs#)	HGVS Name	Chr: Position	Contig	Contig Pos	SNP to Chr	Allele	MAF/Minor	Map Methods
NCAN								
rs2238675	NM_004386.2: c.1072+529 C>T	19:19225799	NT_011295.12	19165799	Fwd	T	T=0.0960/481 (1000 Genomes)	mapup
rs2228603	NM_004386.2: c.274 C>T	19:19159115	NT_011295.12	19159115	Fwd	C	T=0.0439/220 (1000 Genomes)	mapup
TM6SF2								
rs58542926	NM_001001524.2: c.499 C>T	19: 19208740	NT_011295.12	19208740	Fwd	T	T=0.0667/334 (1000 Genomes)	mapup
rs735273	NN_001001524.2: c.-1387 A>G	19:19214602	NT_011295.12	19214602	Fwd	G	C=0.4655/2331 (1000 Genomes)	mapup
CILP2								
rs16996148	NC_000019.9: g.19658472 G>T	19: 19487663	NT_011295.12	19487663	Fwd	G	T=0.1156/579 (1000 Genomes)	mapup
rs17216525	NC_000019.9: g.19662220 C>T	19: 19491411	NT_011295.12	19491411	Fwd	C	T=0.0815/408 (1000 Genomes)	mapup
PBX4								
rs12610185	NM_025245.2: c.119+7598 C>T	19: 19550913	NT_011295.12	19550913	Fwd	C	T=0.1132/567 (1000 Genomes)	mapup
SUGP1								
rs10401969	NM_172231.3: c.1243+80 A>G	19: 19236909	NT_011295.12	19236909	Fwd	A	G=0.1176/589 (1000 Genomes)	mapup
MAU2								
rs73001065	NM_015329.3: c.1548+296 G>C	19: 19289732	NT_011295.12	19289732	Fwd	G	C=0.0319/160 (1000 Genomes)	mapup
rs8102280	NM_015329.3: c.1155+15 G>A	19: 19284941	NT_011295.12	19284941	Fwd	G	A=0.0333/167 (1000 Genomes)	mapup
rs150268548	NC_000019.9: g.19494483 G>A	19: 19323674	NT_011295.12	19323674	Fwd	G	A=0.0260/130 (1000 Genomes)	mapup
rs968525	NM_015329.3: c.1309-483 C>T	19: 19288406	NT_011295.12	19288406	Rev	C	T=0.3033/1519 (1000 Genomes)	mapup

Supplementary Table 2. The sequences of forward and backward primers of the 19p13.11 gene mutations.

Gene	Primer sequence
NCAN	
rs2238675	TGGAAGAGATAATGCCTCAATTGGC
	GGTAGTGTCCAACTCTCATGAACTTG
rs2228603	TCCAACCCAGGCACACAGGATAT
	GCCACCCTCACGACATTGTC
TM6SF2	
rs58542926	CCCTCCCTTCTTTCTTGTGACA
	CCTGCACCATGGAAGGCAAATA
rs735273	CTGCAGCTGGCACAAATTCTAAC
	CCCGCTTACAAGAAGGCTCATTTTA
CILP2	
rs16996148	CCGATCTCATCATTCACCCATC
	GTCCACCCTAGGGCAAAGGAAG
rs17216525	CAGCCAGGAGGGATAGAAGATACT
	CTTCTTTGAGCTGCACCATTCTG
PBX4	
rs12610185	TGTCAAACAACAAAAACCAACACAATT
	GGGAATTTATGATGTGGAATTTCCCAGA
SUGP1	
rs10401969	ATTGCAATAGGCCCAGCAATTCC
	TTGGAAGGGTCTGACTTCTTTCAC
MAU2	
rs73001065	GCATGGCACTGTTCATCCTATG
	CCCTCAGGTGTCAACACATAGC
rs8102280	CAGTTTGGTCAGACAGGACATG
	GAATGTTCTGGATCTGGTTAGGTACTTAC
rs150268548	GGCAAAATGGGCTGCTTTTCT
	GCCTCAGCCCTAGGACAATG
rs968525	AACCTGTCTCAAAGGAAAAAGAAAGCC
	GGCGTGATCTGACTGATAATTTAGCT
